# Comparative response to PDT with methyl-aminolevulinate and temoporfin in cutaneous and oral squamous cell carcinoma cells

**DOI:** 10.1038/s41598-024-57624-8

**Published:** 2024-03-25

**Authors:** J. Nicolás-Morala, M. Alonso-Juarranz, A. Barahona, S. Terrén, S. Cabezas, F. Falahat, Y. Gilaberte, S. Gonzalez, A. Juarranz, M. Mascaraque

**Affiliations:** 1 Department of Biology, Universidad Autónoma de Madrid, Madrid, Spain; 2 Department of Experimental Dermatology and Skin Biology, Instituto Ramón y Cajal de Investigación Sanitaria, IRYCIS, 28034 Madrid, Spain; 3 Oral and Maxillofacial Surgery Service, Hospital Clínico San Carlos, 28040 Madrid, Spain; 4 Surgery Department, Faculty of Medicine, Universidad Complutense, 28040 Madrid, Spain; 5 Oncology Service, Hospital Clínico San Carlos, 28040 Madrid, Spain; 6 Department of Dermatology, Miguel Servet University Hospital, Instituto Investigación Sanitaria (IIS), Zaragoza, Aragón Spain; 7 Department of Medicine and Medical Specialties, Universidad de Alcalá, Madrid, Spain

**Keywords:** Oral cancer, Skin cancer, Cancer therapy

## Abstract

Cutaneous and Head and Neck squamous cell carcinoma (CSCC, HNSCC) are among the most prevalent cancers. Both types of cancer can be treated with photodynamic therapy (PDT) by using the photosensitizer Temoporfin in HNSCC and the prodrug methyl-aminolevulinate (MAL) in CSCC. However, PDT is not always effective. Therefore, it is mandatory to correctly approach the therapy according to the characteristics of the tumour cells. For this reason, we have used cell lines of CSCC (A431 and SCC13) and HNSCC (HN5 and SCC9). The results obtained indicated that the better response to MAL-PDT was related to its localization in the plasma membrane (A431 and HN5 cells). However, with Temoporfin all cell lines showed lysosome localization, even the most sensitive ones (HN5). The expression of mesenchymal markers and migratory capacity was greater in HNSCC lines compared to CSCC, but no correlation with PDT response was observed. The translocation to the nucleus of β-catenin and GSK3β and the activation of NF-κβ is related to the poor response to PDT in the HNSCC lines. Therefore, we propose that intracellular localization of GSK3β could be a good marker of response to PDT in HNSCC. Although the molecular mechanism of response to PDT needs further elucidation, this work shows that the most MAL-resistant line of CSCC is more sensitive to Temoporfin.

## Introduction

Cancer is the second leading cause of death by disease worldwide. In 2020 alone, 19.3 million new cases were diagnosed, with 9.6 million people dying from cancer^1^. It is estimated that one in five people will develop this pathology during their lifetime^1^. Carcinomas, cancers of epithelial origin, are of highest incidence worldwide, being the most common the squamous cell carcinomas (SCC) of the skin, head and neck, oesophagus, lung, and cervix^2–4^.

Cutaneous squamous cell carcinoma (CSCC) originates in the squamous layer of the epidermis. Its incidence ranges between 0.5–16 per 100,000 inhabitants and is estimated to double in the following decades^5,6^. Histologically, it could be a sequential process, but in the majority of cases, CSCCs can be locally invasive, although they can sometimes metastasize. Their lymph node metastasis rate is around 1–2% and their lethality rate reaches 1.5–4%^7–9^. Its aetiology is multifactorial, highlighting environmental factors such as exposure to ultraviolet (UV) radiation, especially UVB^5,6,10^. Head and neck squamous cell carcinoma (HNSCC) originates from the squamous layer of the oral cavity (the most frequent), pharynx, larynx, nasal cavity and even from the salivary glands. In 2020, 890,000 cases and 450,000 deaths were diagnosed of this type of carcinoma according to GLOBOCAN data^10,11^. The incidence of this type of tumour is estimated to increase to 30% by 2030. HNSCC is very aggressive, able to invade surrounding tissues and presenting a metastasis rate between 25 and 45% and a five-year survival rate of 54%^11,12^. The aetiology is multifactorial, although exposure to toxic substances or exposure to human papillomavirus (HPV) stand out^3,11,13^. HPV is mainly responsible for the increase in incidence in recent years, although it now has a better prognosis^11,14^.

For both, CSCC and HNSCC, treatments are selected according to the stage, the anatomical area, surgical accessibility and, of course, the patient's decision. Classic treatment includes surgery, chemotherapy (5-Fluorouracil in CSCC and cisplatin in HNSCC) or radiotherapy, as well as their combination in selected cases^7,12,14,15^. There are newer treatments for in situ tumours, which are more respectful for the surrounding tissues with less toxicity, such as photodynamic therapy (PDT)^16–19^. PDT is based on the combined action of a photosensitizer (PS), light of a certain wavelength (depending on the photosensitizer) and oxygen. The PS, administered locally or systemically, is excited with a specific light wavelength, generating reactive oxygen species (ROS) responsible for tumour cell death^19–21^. These created ROS can react with different biological molecules such as lipids and nucleic acids, inducing tumour cell death^20,21^. Within this process, PDT also induces an indirect vascular effect of the area generating hypoxia and stimulation of the immune system at the local level^19^. The efficacy of this therapy depends on the PS, its doses and time of administration and the type of light received^16^. PDT has a number of advantages over more common treatments, such as chemotherapy and radiotherapy, since it has low systemic toxicity, shorter treatment time, better cosmetic results and no long-term side effects^3,19–22^. Even so, it has certain disadvantages such as local pain (mild-moderate), transient systemic photosensitivity, or the possibility of developing resistant cells to the therapy, either by repeated exposure or undertreatment.

The compounds approved for PDT in the case of in situ CSCC are: 5-aminolevulinic acid (ALA) and methylaminolevulinate (MAL). Both compounds are PS´s precursors and are topically applied. These precursors generate protoporphyrin IX (PpIX), an intermediate metabolite of the heme group synthesis pathway, which accumulates primarily in tumour cells, due to enzymatic alterations in the heme synthesis pathway^23^. Temoporfin, a PS in itself, is approved for the palliative treatment of HNSCC^16,17^. It is administered intravenously, with a maximum accumulation between 24 and 48 h and requires a low dose of PS and light to generate tumour phototoxicity^24^.

Even though PDT is an excellent option for several subtypes of CSCC and HNSCC, as it happens with other cancer therapies, resistance can occur. Different activated oncogenic signaling pathways as well as tumor microenvironment modulation through cancer-associated fibroblasts and the immune system infiltration, among others, influence basal tumour resistance to PDT^23,25–27^. Tumour resistance to diverse therapies has been correlated with epidermal mesenchymal transition (EMT) in a wide variety of tumours^25–30^. EMT process is responsible for resistance to several treatments as chemotherapy and immunotherapy as well as the elevated metastasis rates^31,32^. EMT consists of a cellular reprograming of importance for physiology and organism maintaining and is characterized by a loss of the epithelial phenotype (E-cadherin, β-catenin membrane expression) and gain of mesenchymal properties (vimentin, N-cadherin, snail and migratory phenotype)^29^. However, in a carcinogenic landscape, this program is activated in the absence of appropriated signalling, leading to the detachment of epithelial tumours cells, extracellular matrix (ECM) remodelling and mesenchymal features acquisition^29–31^.

The alteration of Wnt/β-catenin pathway has been implicated in the EMT as well in resistance to cancer therapies^33–35^. In a physiological situation, β-catenin forms part of the cell junctions together with E-cadherin. Its excess is degraded via proteasome. However, in cancer, the cytoplasmic destruction complex is not formed, β-catenin is not degraded and can translocate to the nucleus where it acts as a transcription factor, activating genes involved in proliferation, invasion, and multidrug resistance^33–35^. One of the proteins involved in the cytoplasmic destruction complex responsible for β-catenin degradation is the serine threonine kinase GSK3β^35–37^. The translocation of GSK3β to the nucleus regulates, among different transcription factors, NF-κβ^38^. NF-κβ promotes tumour progression, cellular metabolic changes, cancer stem cell induction, EMT process and tumour invasion, among others^39–41^. Persistent activation of NF-κβ is widely extended among malignancies^41^. It has also been described that NF-κβ can induce tumour resistance to chemotherapy and even promote metastasis^42^.

According to the above, it is necessary to correctly approach the treatment of CSCC and HNSCC. Although completely eradicating the tumour is the fundamental objective of treatments, this is not always possible due to resistance processes. Therefore, the objective of this work is to compare the response to PDT with two clinically employed PSs, Temoporfin and MAL, of CSCC and HNSCC lines and to establish a relationship with the EMT process and the activation of the GSK3β/ NFκβ pathway, in order to acquire a better knowledge of the response to the therapy and selecting the most appropriate photosensitizer for each tumour.

## Results

### Proliferative, migratory and EMT evaluation of cutaneous and head and neck squamous cell carcinoma cell lines

**Figure 1:**
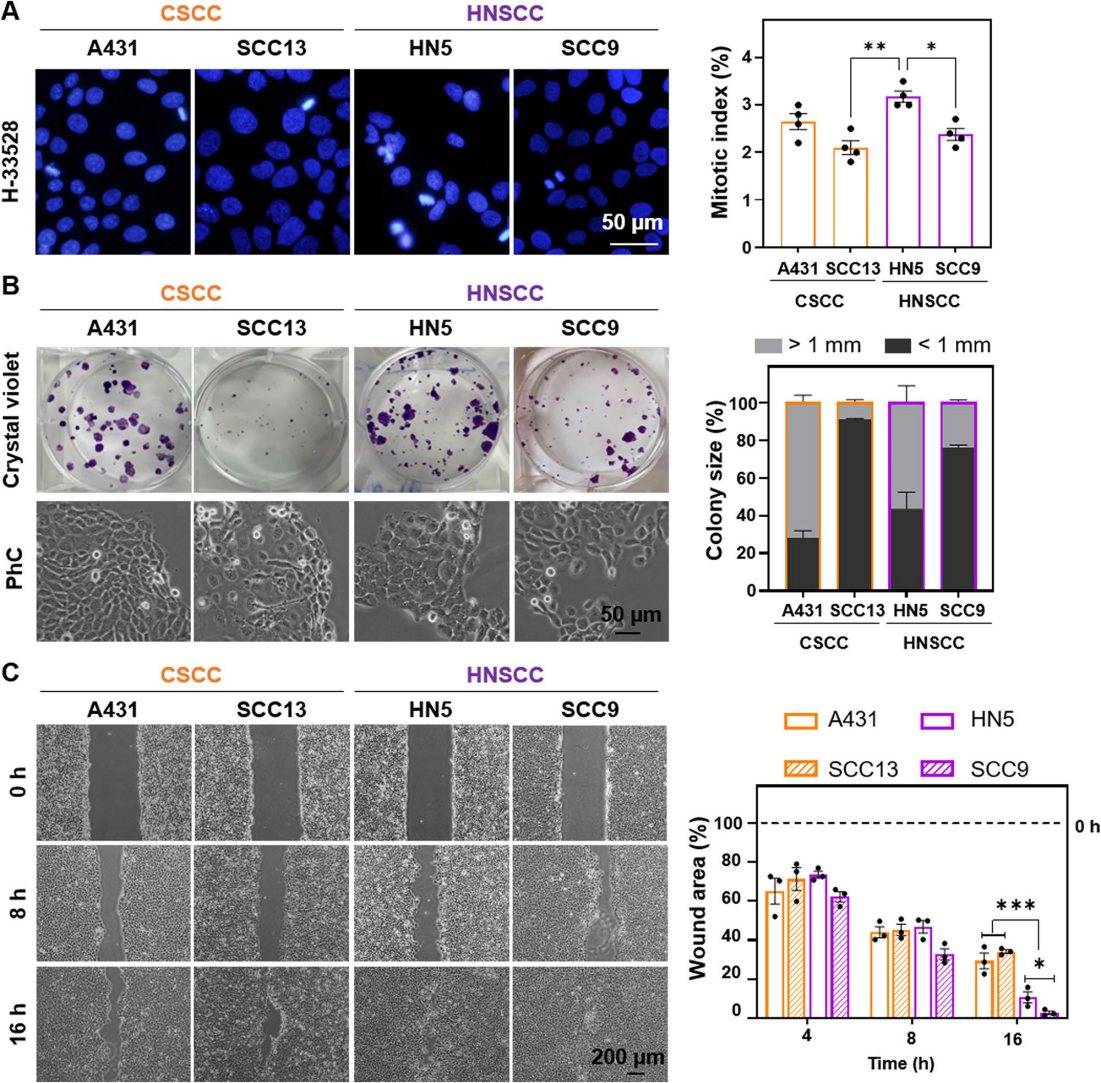
Cell proliferation and migration in CSCC and HNSCC. (**A**) Nuclei (blue) were stained with Höechst-33258 and observed by fluorescence microscopy under ultraviolet excitation light. Mitotic index of cell lines was estimated by the number of dividing cells/total cell number. At least 500 cells of each cell type were estimated. Photographs (left) and quantification (right), n = 4. (**B**) 100 cells per well were seeded, after 14 days of growth, the colonies formed were fixed and stained with crystal violet. Colonies were classified according to their size into < 1 mm and > 1 mm. Photographs (left) and quantification (right), n = 3. (**C**) Wound closure evolution after inserts removal at 0 h. Photographs were taken at 0, 8 and 16 h (left panel) and quantification of the cell-free area (right panel), n = 3. Values are represented as the mean ± SEM (*p < 0.05, **p < 0.01, ***p < 0.001).

The proliferative capacity of the different cell lines (A431 and SCC13 of CSCC and HN5 and SCC9 of HNSCC) was assessed by estimating the mitotic index and colony assay (Fig. 1A,B). The HN5 cell line showed the highest proliferative rate (highest mitotic index) (Fig. 1A) and 60% of colonies formed larger than 1 mm in size (Fig. 1B). SCC13 and SCC9 showed the lowest proliferative rate. These results indicate that there is no significant difference at the proliferative level between the CSCC and HNSCC lines. In addition, colonies formed by HNSCC cell lines were less compact than those formed by CSCC cell lines, as the margins of the colonies show cells with a more elongated morphology (Fig. 1B). Based on this lower degree of compaction, we performed a migration test using a wound healing assay. HNSCC cells (HN5 and SCC9) closed the wound at a faster rate than CSCC cells (A431 and SCC13). SCC9 was the most migratory cell line (Fig. 1C).

**Figure 2:**
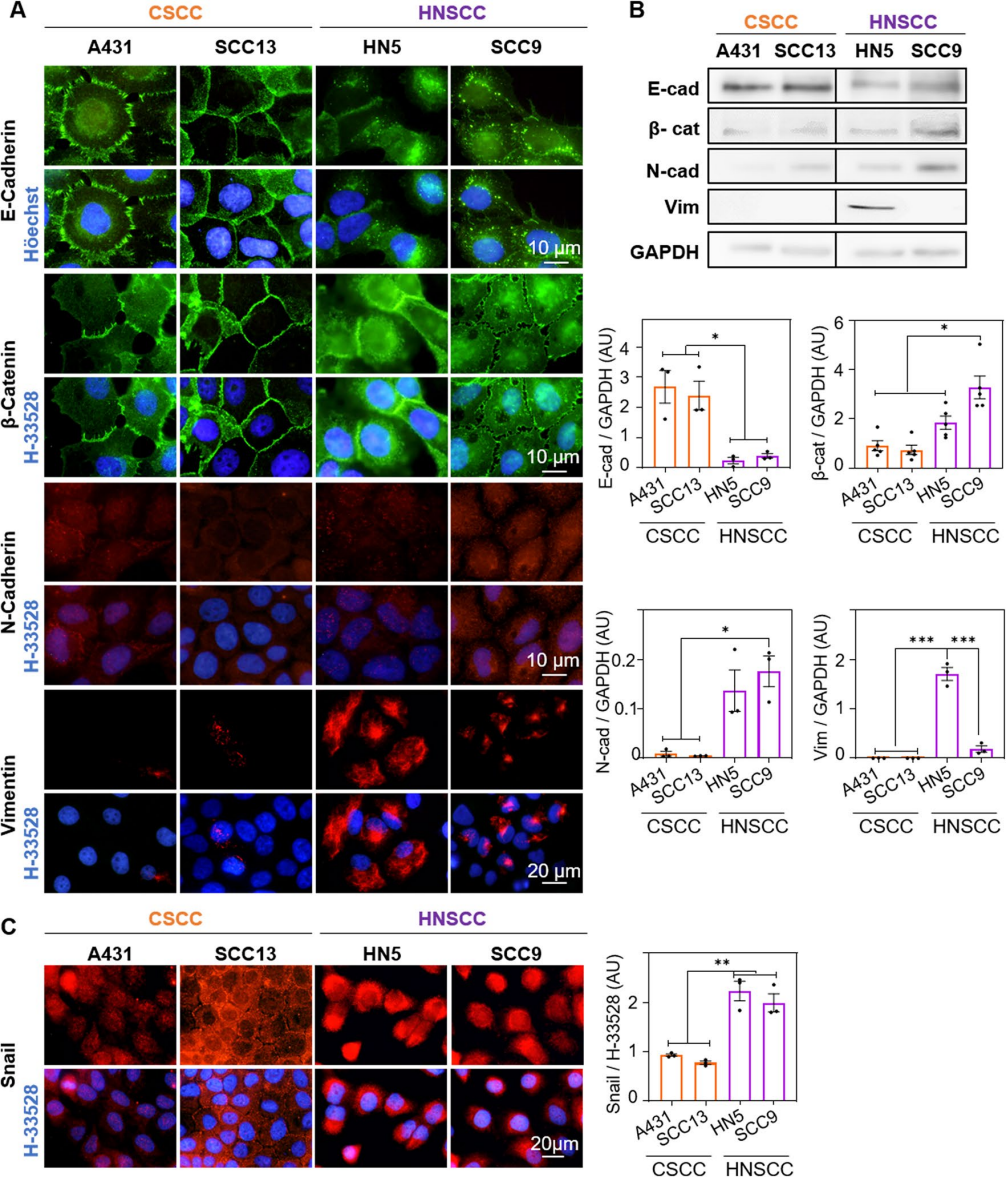
EMT markers in CSCC and HNSCC. (**A**) Localization of E-cadherin, β-catenin, N-cadherin and vimentin assessed by indirect immunofluorescence. Nuclei are counterstained with Höechst-33258 (blue), n = 3. (**B**) Quantification of E-cadherin, β-catenin, N-cadherin and vimentin expression by Western Blot. A representative expression band and the densitometry of these bands relative to the loading control (GAPDH) are shown, n = 3. (**C**) Localization of Snail (red) determined by indirect immunofluorescence. Nuclei are counterstained with Höechst-33258 (blue) (left panel) and Snail expression by quantification of fluorescence intensity (right panel), n = 3. Values were represented as mean ± SEM (*p < 0.05, **p < 0.01, ***p < 0.001).

Based on the migratory phenotype differences observed in the wound healing assay between the cell lines, we then proceeded to analyse the expression of several markers involved in the EMT. The expression and localization of two epithelial markers (E-cadherin, β-catenin) and three mesenchymal markers (N-cadherin, vimentin, snail) were analysed by indirect immunofluorescence (Fig. 2A) and Western Blot (Fig. 2B, Fig. Supp. 1A). E-cadherin was mostly located at the plasma membrane level in both CSCC lines, while HNSCC lines presented a more heterogeneous distribution, being lost at the membrane level and diffusely expressed at the cytoplasmic level (Fig. 2A). Likewise, Western Blot quantification showed significantly higher amounts of E-cadherin in CSCC compared to HNSCC lines (Fig. 2B). Regarding β-catenin, it was located in the plasma membrane in CSCC cell lines, whereas in the HN5 line, some heterogeneity was observed, being β-catenin localized mostly at the cytoplasmic level, while in SCC9 it was found both at the cytoplasmic and nuclear level (Fig. 2A). Likewise, it was observed that SCC9 line was the one with the highest β-catenin expression, when compared to the rest of the cell lines (Fig. 2B). Additionally, the expression of mesenchymal proteins, N-cadherin and vimentin were found to be diffusely localized in the cytoplasm of all cell lines, although with a higher expression in HNSCC cells. The expression of N-cadherin was significantly higher in the SCC9 and vimentin in the HN5 cell line (Fig. 2A,B). Finally, snail expression was assessed by indirect immunofluorescence. It was observed that the SCC9 line showed the highest expression of this marker, being located at the nuclear level. In addition, in terms of total expression (quantified by fluorescence intensity with Image J), HNSCC lines showed significantly higher levels of snail than CSCC ones (Fig. 2C). Therefore, the two CSCC cell lines are less migratory and possess fewer mesenchymal markers than the HNSCC lines.

**Figure 3:**
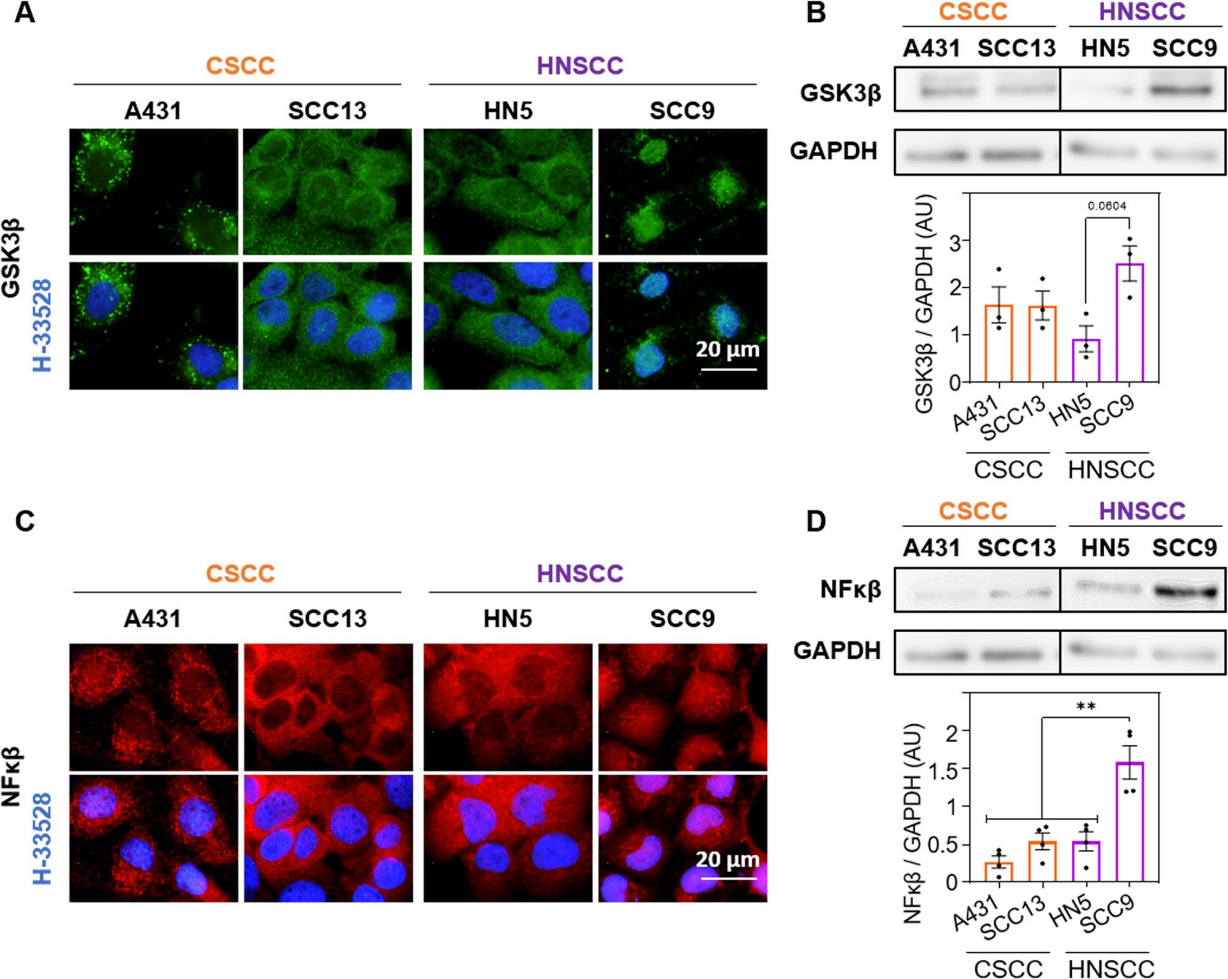
GSK3β and NF-κβ expression. (**A**) Localization of GSK3β (green) determined by indirect immunofluorescence. Nuclei are counterstained with Höechst-33258 (blue), n = 3. (**B**) Quantification of GSK3β expression by Western blot. A representative band and densitometry of the bands relative to the loading control (GAPDH) are shown, n = 3. (**C**) Localization of NF-κβ (red) determined by indirect immunofluorescence. Nuclei are counterstained with Höechst-33258 (blue), n = 3. (**D**) Expression and quantification of NF-κβ by Western blot. Representative band and densitometry of these relative to the loading control (GAPDH) are shown, n = 3. Values are represented as mean ± SEM (**p < 0.01).

After a preliminary cell line characterisation, we assessed the expression and localization of elements of the GSK3β/NF-κβ pathway, which is related to β-catenin. In the CSCC cells, GSK3β was located in the cytoplasm, while in HNSCC presented a differential localization: in HN5 was situated in the cytoplasm meanwhile in SCC9, GSK3β was observed at nuclear level (Fig. 3A). In addition, the expression of this molecule was higher, although not statistically significantly, in the SCC9 cells (Fig. 3B, Fig. Supp 1B). Since, it has been described that the nuclear localization of GSK3β is capable of activating the transcription factor NF-κβ, we proceeded to evaluate the localization and expression of the later in the different cell lines. We observed the same trend as in GSK3β, NF-κβ was located in the cytoplasm of A431, SCC13 and HN5 and in the nuclei on the cell line SCC9 (Fig. 3C). The results obtained by Western Blot indicated that the expression of NF-κβ was significantly higher in SCC9 compared to the rest of the cell lines (Fig. 3D).

### Photodynamic therapy with methyl-aminolevulinate and Temoporfin

After comparing the functional behaviour (proliferation and migration) and expression of markers of EMT and GSK3β/NF-κβ pathway of each of the cell lines, we proceeded to evaluate their response to PDT. For this, we used two compounds, MAL and Temoporfin, which are being used for treatment of certain types of CSCC and HNSCC, respectively. The response to PDT of the cell lines was evaluated by MTT assay 24 h after treatments. In a first approach, the toxicity was analysed in the absence of light, of the compounds used, MAL (5 h, 0.5 mM) and Temoporfin (24 h, 25 nM). The concentrations of the compounds and light dose were selected based on previous results^43,44^. The results obtained indicated that, under such experimental conditions, neither the compounds nor the red light by themselves, caused cytotoxic damage to the cells (Table 1).

**Table 1:** Absence of toxicity of the MAL, Temoporfin and red light independently administered to the cultures.

	A431	SCC13	HN5	SCC9
MAL [0.5 mM]	99.3 ± 1.9	100.6 ± 1.5	101.8 ± 3.2	100.0 ± 3.1
Temoporfin [25 nM]	99.9 ± 2.7	100.2 ± 0.9	102.7 ± 1.0	100.1 ± 0.9
Red light (12 J/cm^2^)	100.1 ± 3.5	100.1 ± 0.3	99.1 ± 2.5	102.1 ± 2.6

The values are the results obtained from the MTT assay relative to untreated control cells, n = 3. Values were represented as mean ± SEM.

**Figure 4:**
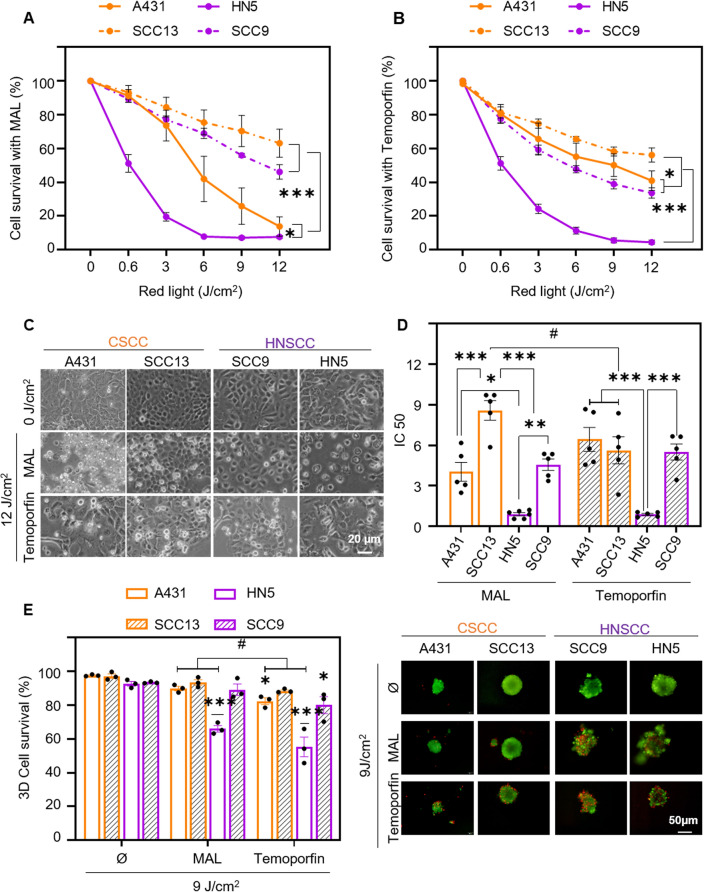
Photodynamic therapy with methyl-aminolevulinate and Temoporfin. Cell survival was determined by MTT assay 24 h after incubation with 0.5 mM MAL for 5 h (**A**) or 24 h with 25 nM Temoporfin (**B**) and subsequent irradiation with red light (0 to 12 J/cm^2^). The results of the MTT assay are relativized to the values of absorbance at 542 nm obtained for untreated cells (indicated as control), n = 5. (**C**) Cell morphology after PDT (5 h of MAL or 24 h Temoporfin incubation followed by 12 J/cm^2^ dose) and observed by phase contrast microscopy 24 h after irradiation. (**D**) Half maximal inhibitory concentration (IC50) is represented for both treatments in each cell line, n = 5. (**E**) Cell survival after PDT (0.5 mM MAL, 9 J/cm^2^ or 25 nM Temoporfin, 9 J/cm^2^) in spheroids. Quantification of cell survival was determined by staining with acridine orange and propidium iodide and estimating the green (live) cells with respect to red (dead) cells, n = 3. Values were represented as mean ± SEM (*p < 0.05, **p < 0.01, ***p < 0.001, #*p* < 0.05 MAL vs Temoporfin).

The response to PDT with MAL was then assessed. For this purpose, cells were incubated with the compound for 5 h and then exposed to a range of red light doses from 0.6 to 12 J/cm^2^. The results obtained indicated that cell lethality was light dose-dependent in all cell lines. Likewise, the HN5 cell line was the most sensitive, followed by A431, being the most resistant cells SCC13 and SCC9 (Fig. 4A). In the case of Temoporfin, cells were incubated for 24 h at a concentration of 25 nM and then exposed to variable doses of light. In this case, HNSCC lines were significantly more sensitive than CSCC lines; HN5 was also the most sensitive cell line to this treatment (Fig. 4B). Figure 4C shows the morphological changes caused by MAL and Temoporfin treatments at the highest light dose (12 J/cm^2^). The IC50 of both treatments was calculated for each cell line and correlated with the dose–response curves in Fig. 4A,B. SCC9, HN5 and A431 cell lines exhibited no significant differences in the IC50s of both treatments. In contrast SCC13 cell line reduced significantly the required light dose to achieve an IC50s when treated with Temoporfin (6 J/cm^2^), requiring a higher light dose when MAL is employed to reach a IC50s (almost 9 J/cm^2^) (Fig. 4D). In parallel, the same treatments were assessed in a three-dimensional spheroid model after 9 J/cm^2^; this fluence was selected considering that it is the dose above the IC50 for all 2D conditions keeping in mind that spheroids need higher PDT fluences than monolayer cultures. In this case, viability was evaluated by staining with propidium iodide and acridine orange, where dead cells fluoresce red and live cells fluoresce green. 3D models, better PDT efficacy was observed with Temoporfin than with MAL in most cell lines (Fig. 4E).

### Subcellular localization of photosensitizer and ROS production

**Figure 5:**
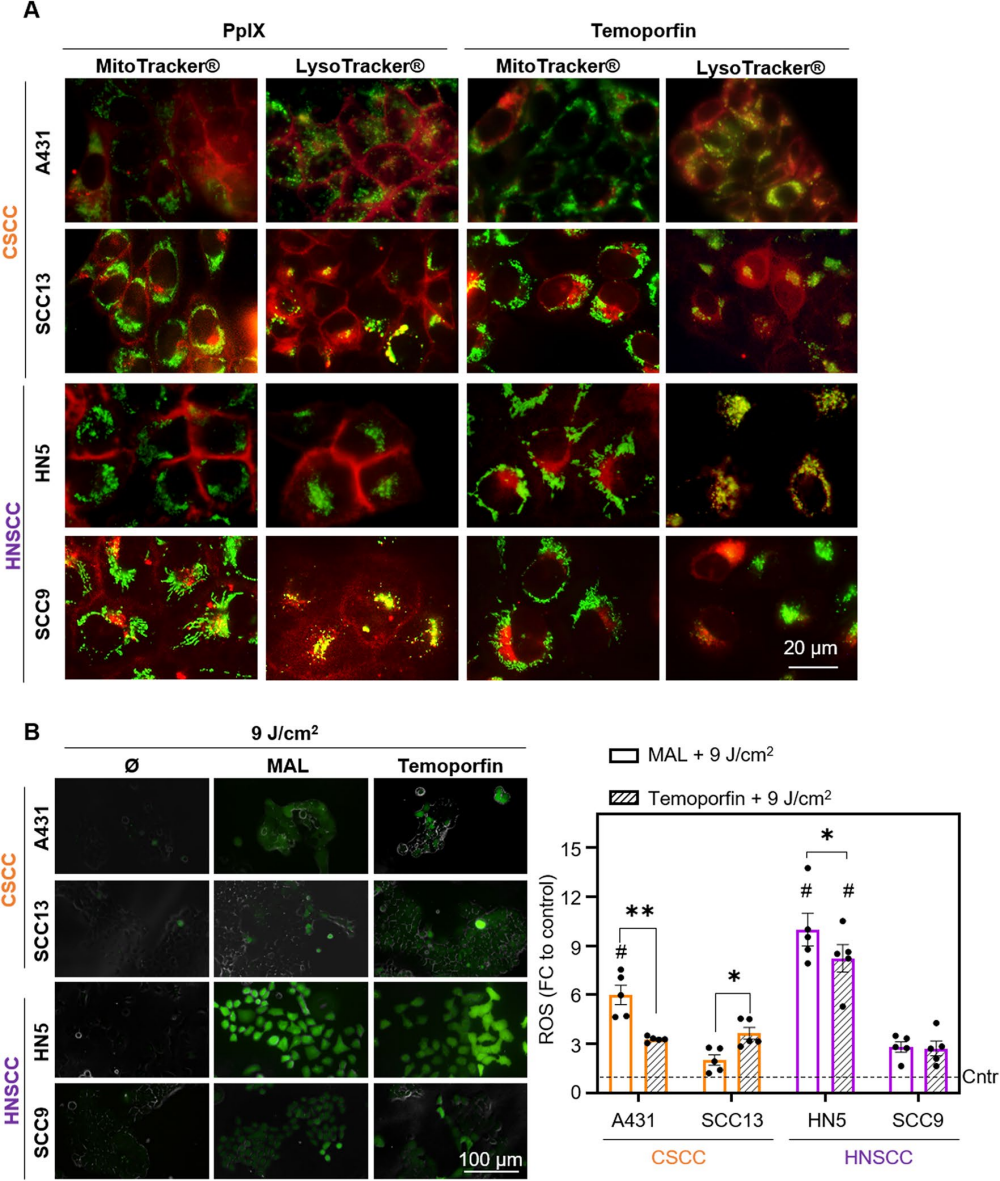
Subcellular localization of photosensitizer and ROS production. (**A**) Cells were incubated with MAL (0.5 mM for 24 h) and with Temoporfin (25 nM for 24 h) and the localization of PS (red fluorescence) determined by fluorescence microscopy. Green fluorescence caused by MitoTracker® (mitochondria) or LysoTracker® (lysosomes) probes, n = 3. (**B**) ROS production detected by the DHF-DA fluorescent probe after PDT with MAL or Temoporfin and red light (9 J/cm^2^). Cells were incubated MAL (0.5 mM for 24 h) and with Temoporfin (25 nM for 24 h), and in the last hour DHF-DA was added, reaching a final concentration of 6 μM. The fluorescence signal was observed by using fluorescence microscopy (λexc = 436 nm). Intracellular fluorescence intensity was measured by ImageJ, n = 5. Values were represented as mean ± SEM (*p < 0.05, **p < 0.01, ***p < 0.001, #*p* < 0.05 different cells between same treatment).

Since the intracellular localization of PSs could be related to the response to PDT, we proceeded to study the subcellular localization of PpIX (endogenous PS formed after MAL administration) and Temoporfin by fluorescence microscopy. To this end, a co-localization study was performed with organelle-specific markers, MitoTracker® (mitochondria) and LysoTracker® (lysosomes). In the case of PpIX, none of the four lines showed red fluorescence, due to PpIX, at the mitochondrial level. In both CSCC cells, PpIX was localized at the plasma membrane and also, in SCC13 cell line at the lysosomes. In HN5 cells, red fluorescence of PpIX was only observed at the plasma membrane level and, in SCC9 cells, it was preferentially localized in the lysosomes (showing yellow fluorescence after superimposing the images) and, to a lesser extent, in the cell membrane (Fig. 5A, Fig. Supp 2). In the case of Temoporfin, the red fluorescence was observed in the lysosomes, co-localizing with the signal produced by the LysoTracker® marker; a yellow fluorescence was observed after superimposing the images confirming such colocalization (Fig. 5A, Fig. Supp 3).

Finally, we have further evaluated the production of ROS by using the fluorescent ROS sensor DHF-DA after the different conditions of PDT applied to the cells. The results obtained showed the highest increase in ROS after PDT with both MAL and Temoporfin in the HN5 cell line, comparing to the rest of the cells; this result is consistent with the previously obtained MTT results that showed that HN5 cell line was the most sensitive to PDT. In addition, it was observed that comparing both treatments, MAL PDT produces higher levels of ROS than Temoporfin only in A431 and HN5 cell lines, whereas in SCC13 cells Temoporfin induced-ROS levels were higher than MAL-PDT. No significant differences were appreciated in SCC9 (Fig. 5B).

## Discussion

SCCs are the most common type of solid cancers, and their incidence continues increasing nowadays. Among SCC multiple types, CSCC and HNSCC stand out. PDT importance on the therapeutic landscape of these tumours resides on the selectivity, which is essential considering their anatomical locations^18,44,45^. In this work we have analysed the response to MAL and Temoporfin as PSs for PDT in cutaneous and oral squamous carcinoma cells (CSCCs and HNSCs) and aimed to find a correlation with the EMT process and the activation of the GSK3β/ NF-κβ pathway. Our results revealed a differential response to treatment depending on the cell line. The CSCC cell line A431 and, surprisingly, the HNSCC cell line HN5 were the most sensitive cells to MAL-PDT. The latter is resistant to cisplatin, a chemotherapeutic compound. Published results on lines resistant to chemotherapeutics (5-FU) showing increased sensitivity to PDT^13,43,46^. As indicated before, Temoporfin is used for the palliative treatment of HNSCC^46^. As expected, HNSCC lines were significantly more sensitive than CSCC lines, with HN5 being the most sensitive cell line to this treatment. Interestingly, although most cell lines showed no significant differences between the two treatments, the SCC13 cell line showed a higher PDT susceptibility when treated with Temoporfin, opening the possibility of a new therapeutic window for the most resistant CSCCs.

To understand the differential response to PDT, we characterized the lines in terms of proliferative capacity and EMT markers expression. In the first case, we observed that A431 and HN5, the most sensitive cell lines to MAL, exhibited the highest mitotic index and formed colonies of greater diameter. However, although it was clearly observed that both HNSCC lines showed more mesenchymal characteristics than CSCC lines (higher migration rate, reduced E-cadherin expression and increased mesenchymal markers N-cadherin, vimentin and snail), which has been associated with increased resistance to various treatments, no relationship with PDT efficacy was observed in our case^47–49^.

In CSCC, E-Cadherin, involved in cell–cell adhesion, was located at the cell membrane, and whereas in HNSCC cell lines was present dispersed in the cytoplasm. A decrease in the membrane expression of E-cadherin is considered an unfavourable prognostic factor, as it decreases during dissemination, invasiveness and relapse in many type of cancers, including non-small cell lung cancer and prostate cancer^47^. In addition, lower expression of this molecule correlates with bad response to PDT^48,49^. The results obtained indicated that E-cadherin expression was significantly higher in CSCC than in HNSCC, correlating with invasion and metastasis rates, supporting results already published^50^. Other epithelial marker analysed was β-catenin, which was distributed at the cell membrane level in CSCC, participating with E-cadherin in the intercellular unions. However, in SCC9 cells, β-catenin was mainly located at the nuclear level. Nuclear level localization of β-catenin has been related to the expression of genes involved in EMT, invasion, multidrug resistance and stem cell generation in human tumors^33–35,51^. Likewise, N-cadherin expression has been associated with differentiation stage, invasion, metastasis and with resistance to Gefitinib^50,52^. According to the literature, an increase in N-cadherin expression is usually accompanied by the loss of E-cadherin, coinciding with our results^52^. Also, high vimentin expression has been correlated with tumour growth, invasion, motility, directional migration and increased cell stiffness in breast, prostate, lung and melanoma cancers^53^. Furthermore, in ovarian cancer cells, its expression has been linked to cisplatin resistance^54^. We have observed that HN5, which was obtained from a patient who received chemotherapy and radiotherapy without favourable results, was the cell line that presented the highest expression of this molecule. One of the transcription factors that initiate the EMT process is snail. The expression of this molecule has been correlated in breast cancer with recurrence, poor survival and malignancy, as well as decreased expression of E-cadherin and increased expression of vimentin as well as by the expression of β-catenin at the nuclear level that stimulates cell migration^33,48,50^. In this context, cells that presented a more mesenchymal phenotype (SCC9 and HN5) showed a higher expression of this molecule^55^.

**Figure 6:**
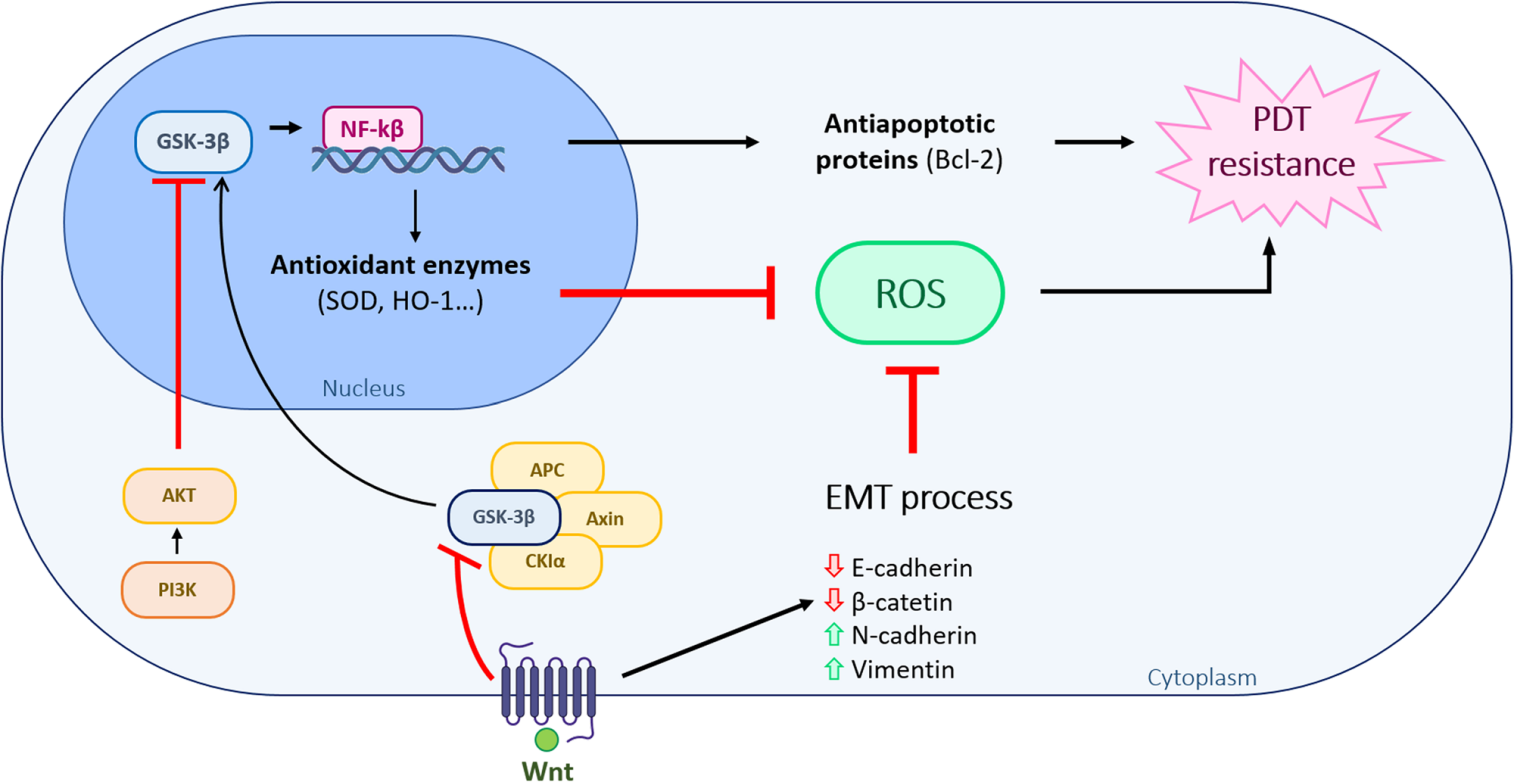
Schematic relation between EMT and the GSK3β/NF-κβ pathway and PDT resistance. Abnormal expression of Wnt/β-catenin pathway or loss of phosphatidylinositol 3-kinase (PI3K)-Akt signalling is related to the nuclear localization of GSK3β. GSK3β nuclear translocation constitutes an upstream regulator of nuclear NF-kB, functioning as a transcription activating diverse antiapoptotic and antioxidant target enzymes. One of the mechanisms through which EMT can induce PDT resistance is the ability to provoke low quantities of ROS. This reduction in ROS production can be also produced by other signalling pathways as the activation of pro-survival signals as a result of NF-kB transcriptional activities.

As we have observed a nuclear localization of β-catenin in the SCC9 line, we proceeded to evaluate the expression of GSK3β/NF-κβ. The nuclear localization of GSK3β has been related to a mesenchymal phenotype in triple negative breast cancer and pancreatic cancer, and the increase in its expression with poor prognosis in urothelial carcinoma renal, pancreatic, leukemia and triple negative breast cancer^38^. Furthermore, in pancreatic carcinoma it has been related to resistance to radiotherapy and chemotherapy, since it is capable of activating the nuclear transcription factor NF-κβ, involved in proliferation and survival^56^. The nuclear localization of GSK3β could be related to intrinsic tumour features and aberrant expression of different signalling pathways as Wnt/β-catenin or loss of phosphatidylinositol 3-kinase (PI3K)-Akt signalling^57^. GSK3β nuclear localization seems to also regulate nuclear NF-kB localization, acting as a transcription factor promoting the transcription of different antiapoptotic and antioxidant proteins, including superoxide dismutase, NAD(P)H dehydrogenase [quinone]1, heme oxygenase-1 and glutathione peroxidase-1^58^ (Fig. 6). The obtained results have shown a differential localization of this molecule; cytoplasmic in both CSCC and in HN5, the cell line most sensitive to treatment with MAL-PDT, and at nuclear level in SCC9. In SCC9, the nuclear expression of GSK3β coexists with that of β-catenin. It has been determined that this co-localization neither alters the subcellular distribution of β-catenin, nor modifies its DNA binding capacity^59^.

We have also evaluated the PSs localization to check if could be related to PDT resistance. We did not observe PpIX mitochondrial localization in any of the cell lines, in concordance with other authors^60^. However, we did appreciate the localization in the lysosomal compartment in SCC13 and SCC9, the most resistant cells to MAL-PDT^61^. As other authors have pointed, lysosomal accumulation of PpIXs produced a limited damage after irradiation, supporting the differential response observed when the cell lines were treated with PDT^62^. However, the lines that display more sensitivity to MAL-PDT, HN5 and A431, only showed localization of PpIX at the plasma membrane. Temoporfin accumulated in the different lines in the lysosomal compartment. Temoporfin that localizes with the lysosomes has been suggested to be retained in the lysosomal compartment, limiting the PDT induced damage^63^. The mechanism through which PS subcellular localization could influence on PDT resistance on those cell lines still remains unclear. Specific studies are needed to clarify the role of PS lysosomal accumulation of CSCC and HNSCC.

As observed in the obtained results, ROS production after PDT with MAL and Temoporfin was elevated on those cells that were more sensitive, as other authors have previously described^64,65^. ROS assay is a feasible measurement to predict PDT induced damage^66^. However, several factors can be modulating PDT effectivity; differential accumulation of the PS, its subcellular localization and the cell metabolism to trigger the antioxidant response. This antioxidant response will also play an important role on ROS-induced cell death and cancer progression^63,67^. One of the mechanisms through which EMT can induce therapy resistance is the ability to created low quantities of ROS^63^. This low ROS production, responsible of PDT resistance, can be modulated by, among others, the activation of pro-survival signals resulting on NF-kB transcriptional activities (Fig. 6). This seems to be the case of the SCC9 cell lines, which presents EMT features and displayed the highest PDT resistance.

From this study it can be concluded that HNSCC cell lines present a more advanced EMT program, higher expression of mesenchymal markers and lower expression of epithelial markers. But this difference in EMT marker expression is not related to the response to PDT either with MAL or Temoporfin. However, we have observed that the most sensitive lines to MAL-PDT are those that presented a higher proliferative index and PpIX located at the plasma membrane. In addition, the differential response to MAL-PDT in HNSCC could be also related to the signalling cascade triggered by the nuclear localization of GSK3β by regulating the localization and expression of the transcription factor NF-κβ, related to resistance against a variety of antitumoral treatments. Therefore, we propose that intracellular localization of GSK3β is a good marker of response to PDT in HNSCC. On the other hand, the most resistant CSCC line, SCC13, is more sensitive to Temoporfin than to MAL-PDT. However,, further studies are needed to optimize PDT for HNSCC and CSCC and select the most appropriate PS according to their cellular and molecular characteristics.

## Materials and methods

### Cell cultures

The CSCC used were A431^68^ (from American Type Culture Collection (Manassas, VA, USA)) and SCC13^69^ (kindly provided by Dr. J.G. Rheinwald Boston, MA, USA) and the HNSCC cell lines used were HN5 and SCC9^45,70^ (kindly provided by Dr. A. Sastre (Research Institute Hospital Universitario La Paz, Spain)). Two-dimensional (2D) cultures were grown in DMEM (Dulbecco’s modified Eagle’s medium high glucose) supplemented with 10% (v/v) fetal bovine serum (FBS) and 1% antibiotic (penicillin, 100 units/mL; streptomycin 100 mg/mL), all obtained from Thermo Fisher Scientific Inc (Rockford, IL, USA). Cell cultures were performed under standard conditions of 5% CO_2_, 95% humidity, and 37 °C and propagated by treatment with 1 mM EDTA/0.25% Trypsin (w/v). For the formation of spheroid (3D cultures), cells were trypsinized, centrifuged at 1800 rpm for 5 min and resuspended in spheroids medium [DMEM/ F12 (1:1), 2% supplement B27 (Thermo Fisher Scientific Inc, Rockford, IL, USA), 20 ng/mL EGF, 0.4% bovine serum albumin and 4 mg/ mL insulin (Sigma-Aldrich, St. Louis, MO, USA)]. Then, cells were seeded at a density of 40.000 cells/mL on P6 plates covered with 1.2% poly-HEMA (2-hydroxyethyl methacrylate, Sigma-Aldrich) in 95% ethanol^45^. The number and diameter of spheroids was determined 5 and 10 days after the cell seeding, using an inverted microscope and the Image J program (NIH, USA).

### Indirect immunofluorescence

The cells cultured on glass coverslips were fixed at the indicated time points after the treatments (see supplementary Fig. 1). Fixation was performed with 3.7% formaldehyde in PBS and permeabilization with 0.5% Triton-X-100 (Merck) in PBS, each step for 30 min at 4 °C. After fixation, the samples were blocked with 2% bovine serum albumin (BSA, Sigma) for 30 min at 37 °C and immediately incubated for 1 h at 37 °C with the primary antibodies (E-cadherin, β-catenin (BD Transduction Laboratories), N-cadherin, Vimentin, GSK3β (Abcam), NF-κβ (Cell Signaling) and Snail (Invitrogen)). Then, cells were washed with PBS and incubated with the corresponding secondary antibodies. Nuclear counterstaining was performed with 0.2 μg/ml Höechst-33258 (H-33528) in distilled water, for 5 min at RT. The samples were then washed and mounted with Prolong™ Gold reagent (Life Technologies). Mitotic index was determined by counting cells in division divided by total cells.

### Cell proliferation

Cell proliferation was determined by the clonogenic assay. Cells were seeded at 100 cell/mL per well in P6 plates and grown for 14 days. Then, the cells were fixed and stained with 0.2% crystal violet (Sigma-Aldrich, St. Louis, MO, USA) in 2% ethanol in distilled water for 20 min under constant shaking at room temperature. Finally, the plates were washed with PBS (phosphate buffered saline), air dried and colonies were counted and classified in groups according to their diameter as: small (< 1 mm) and large (> 1 mm).

### Migration assay

A total of 40.000 cells were added to each insert (Ibidi) situated in a plastic plate with complete medium. When cells reached a confluence of 95–100% the insert was extracted, allowing the cells to move to close the wound. Photographs were taken at 0, 4, 8, 16, 24 h, and when wounds were closed. The wound widths were measured employing Image J program (NIH, USA).

### Western blot

For Western blot analysis, cells were lysed in RIPA buffer (150 mM NaCl, 1% Triton X-100, 1% deoxycholate, 0.1% SDS, 10 mM Tris–HCl pH 7.2, 5 mM EDTA), containing the appropriate concentration of Phosphatase Cocktail and Protease Inhibitor Cocktail (Sigma-Aldrich). Protein concentration was measured by the BCA Protein Assay Kit (Termo Scientific Pierce, Rockford, IL, USA). The proteins were electrophoresed and blotted on Immobilon-P PVDF membranes (Millipore Co., MA, USA). Membranes were blocked in PBS-tween 0.1% with 5% non-fat dried milk for 1 h at 25 °C and then incubated with the first antibody overnight at 4 °C (E-cadherin, β-catenin (BD Transduction Laboratories), N-cadherin, Vimentin, GSK3β, GAPDH (Abcam) and NF-κβ (Cell Signaling)). After washing with PBS-tween 0.1%, membranes were subjected to the peroxidase-conjugated secondary antibody and developed by chemiluminescence (ECL, Amersham Pharmacia Biotech, Little Chalfont, UK) employing the high-definition system ChemiDocTR XRS + (Bio-Rad Laboratories, Hercules, CA, USA). The bands corresponding to the different proteins were digitalized employing the Image Lab version 3.0.1 (Bio-Rad Laboratories).

### Photodynamic therapy

The treatments were carried out when the two-dimensional cultures reached 60–70% confluence or the spheroids had a size around 300 µm. For PDT, two compound were used, methyl-aminolevulinate (MAL) (Sigma-Aldrich, St. Louis, MO, USA) and meso-Tetrahydroxyphenylchlorin (m-THPC, Temoporfin) (Sigma). The two-dimensional cultures and spheroids were incubated with 0.5 mM MAL or with 25 nM Temoporfin, both in corresponding mediums without FBS for 5 h or 24 h, respectively and in the dark. Then, the cultures were irradiated at variable light doses (1.5–12 J/cm^2^) by using a red-light emitting diode source (WP7143 SURC/E Kingbright, Los Angeles, CA, USA) with an irradiance of 6.2 mW/cm^2^ and an emission peak of 634 ± 20 nm. To minimize refraction of light, cells were irradiated from the bottom of the culture plates. After irradiation, the medium was replaced by fresh one for 24 h until evaluation.

### Cell viability

To estimate cell survival in the 2D cultures, the colorimetric (3-(4,5-dimethylthiazol-2-yl)-2,5-diphenyl-2H-tetrazolium bromide, MTT) assay was used. To this end, 24 h after the treatments, the culture medium was replaced with 50 μg/ml MTT in DMEM and incubated for 3 h under the usual culture conditions. After that, the medium containing MTT was removed, and the crystalized formazan was dissolved in a DMSO. The optical density at 542 nm wavelength was measured using a plate reader (SpectraFluor, Biotek). In the case of spheroids, cell survival was also evaluated 24 h after irradiation by using the Propidium Iodide (PI)/Acridine Orange (AO) assay, both used at a concentration of 50 μg/mL in PBS. Immediately after adding PI and AO to the cultures, spheroids were analysed under the fluorescence microscope using green (for AO) or red (for PI) exciting light. Survival was determined calculating the green (alive) and red (dead) fluorescence using the ImageJ program (NIH, USA).

### Photosensitizers localization

PpIX and Temoporfin localization was performed by fluorescence microscopy. When cells grown on glass coverslips reached a 70% of confluence, they were incubated for 24 h with MAL (0.5 mM) or Temoporfin (25 nM) diluted in the corresponding medium without FBS. In addition, to compare the subcellular localization, two specific markers were used, for mitochondria (MitoTracker®) and lysosomes (LysoTracker®) (Invitrogen). The markers were incubated with the cells for 5 min at the concentrations indicated by the suppliers. Immediately after, cells were mounted on slides and observed directly under the fluorescence microscope.

### Intracellular ROS

The intracellular production of ROS cells was evaluated as previously described^43^. Cells were incubated with MAL (5 h) or Temoporfin (24 h) and in the last hour 2,7-dichloro-dihydrofluorescein diacetate (DHF-DA, Abcam) was added to the cultures, reaching a final concentration of 6 × 10^−6^ M. Afterwards, and without removing DHF-DA, cells were exposed to red light (9 J/cm^2^) and, immediately after irradiation, analysed by fluorescence microscopy under blue excitation light (λexc = 436 nm). Corresponding controls were performed: cells incubated with DHF-DA without photosensitizers, nor exposed to red light, and cells incubated with the photosensitizers and DHF-DA, but not exposed to red light. ROS production was quantified by using Image J after measuring green fluorescence.

### Microscopy and statistical analyses

Microscopic observations were carried out using an Olympus BX61 epifluorescence microscope, equipped with a HBO 100 W mercury lamp and the corresponding filter sets for fluorescence microscopy: blue (450–490 nm, exciting filter BP 490), and green (545 nm, exciting filter BP 545). Photographs were obtained with a digital camera Olympus DP50 and processed using Adobe PhotoShop CS5 extended version 12.0 software (Adobe Systems Inc., USA). Data were expressed as the mean value of at least three experiments ± standard errors of the mean (SEM). The statistical analysis was carried out with the version 8 of the program GraphPad Prism (GraphPad Software Inc, USA) used, also, to make graphical representations. The statistical differences were determined using, in general, analysis of variance (ANOVA, Chicago, IL, USA) and post hoc Bonferroni's test or Kruskal–Wallis tests, depending on the result of the Shapiro–Wilk normality test; *p* < 0.05 was considered statistically significant. The significant differences were classified as ∗*p* < 0.05; ∗  ∗*p* < 0.01; ∗  ∗  ∗*p* < 0.001.

### Supplementary Information

Supplementary Figures.

## Data Availability

The datasets generated and/or analysed during the current study are not publicly available due they are collected on several hard disks in the laboratory but are available from the corresponding author on reasonable request.
