# Supplementary material for: Comparative response to PDT with methyl-aminolevulinate and temoporfin in cutaneous and oral squamous cell carcinoma cells
Source: Sci Rep. 2024 Mar 25;14:7025. doi: 10.1038/s41598-024-57624-8 (PMC10963768; doi:10.1038/s41598-024-57624-8)
Supplement: Supplementary file 1 — Supplementary Figures. [file 41598_2024_57624_MOESM1_ESM.docx]

**
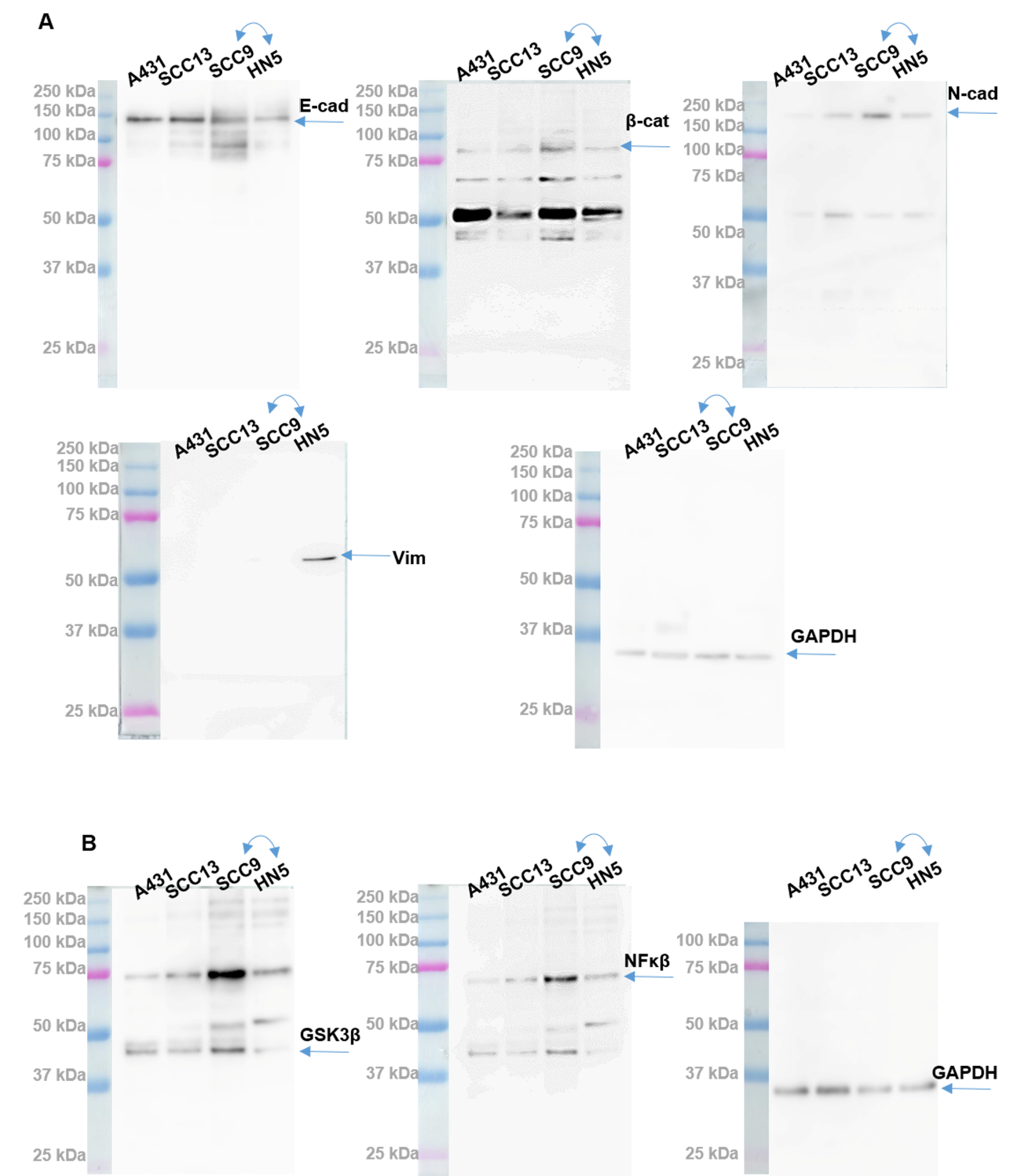
**

**Figure supplementary 1. A)** Full-length blot membranes together with the load marker of Figure 2B and **B)** Figure 3B and D. Note that the order of the extracts changes with respect to the main figures, which were cropped and flipped.

**
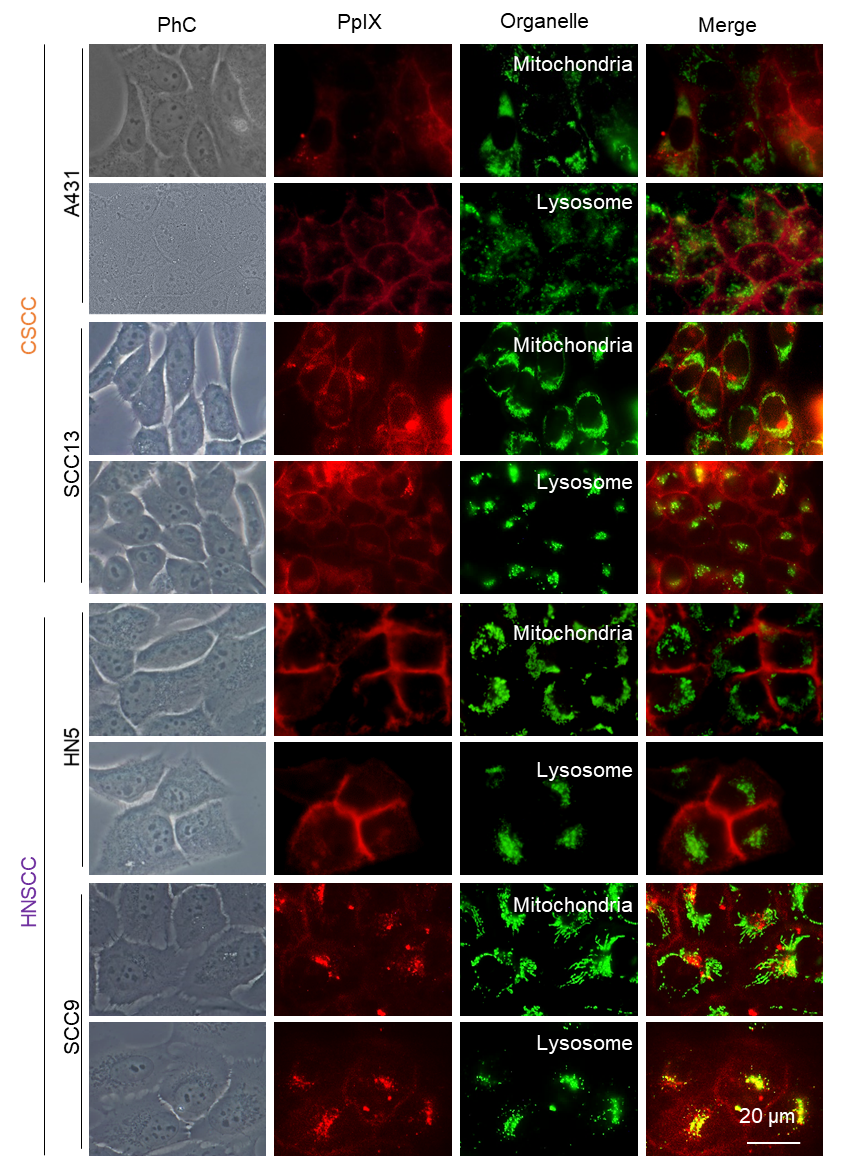
**

**Figure supplementary 2. Subcellular localization of PpIX in the cell lines.** Cells were incubated with MAL (0.5 mM for 24 h) and PpIX production was observed by fluorescence microscopy. PhC: phase contrast, red fluorescence due to PpIX emission under green excitation light, green fluorescence caused by MitoTracker® (mitochondria) or LysoTracker® (lysosomes) probes under blue excitation light. The last column shows the overlay of PpIX localization images with each organelle studied.

**
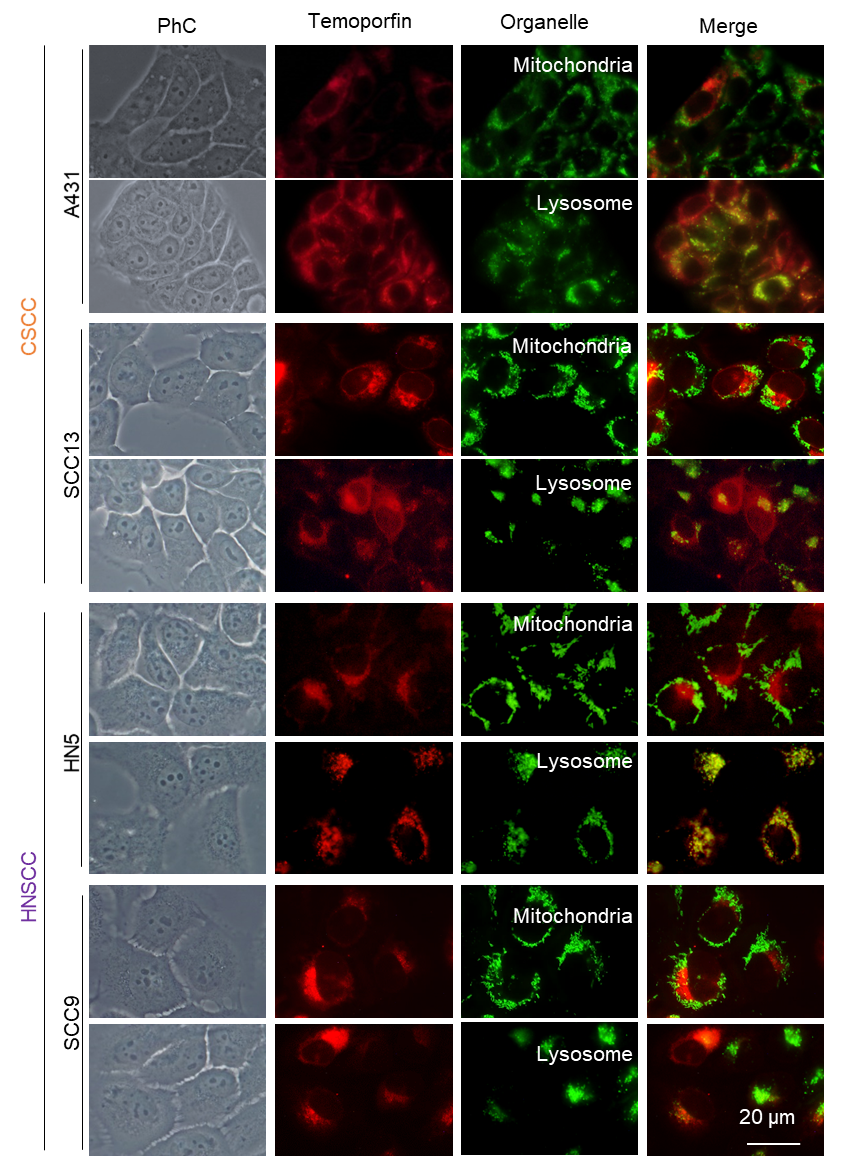
**

**Figure supplementary 3. Subcellular localization of Temoporfin in the cell lines.** Cells were incubated with Temoporfin (25 nM for 24 h) and its localization was observed by fluorescence microscopy. PhC: phase contrast, red fluorescence due to Temoporfin emission under green excitation light, green fluorescence caused by MitoTracker® (mitochondria) or LysoTracker® (lysosomes) probes under blue excitation light. The last column shows the overlay of Temoporfin localization images with each organelle studied.
